# The weakest link in digital atrial fibrillation screening: a randomized comparison of self-applied ECG patch systems in older adults

**DOI:** 10.1093/europace/euag192

**Published:** 2026-08-31

**Authors:** Daniel Pavluk, Benedikt Kindl, Samuel Proell, Patrick Rockenschaub, Theresa Dolejsi, Ivan Lechner, Helen Aumayer, Lena Hofhansel, Konstantinos D Rizas, Dieter Hayn, Guenter Schreier, Martin Manninger, Daniel Scherr, Clemens Dlaska, Sebastian J Reinstadler, Axel Bauer, Michael Schreinlechner

**Affiliations:** University Clinic of Internal Medicine III, Cardiology and Angiology, Medical University Innsbruck, Innsbruck AT-6020, Austria; University Clinic of Internal Medicine III, Cardiology and Angiology, Medical University Innsbruck, Innsbruck AT-6020, Austria; Digital Cardiology Lab, University Clinic of Internal Medicine III, Medical University Innsbruck, Innsbruck AT-6020, Austria; Institute of Clinical Epidemiology, Public Health, Health Economics, Medical Statistics and Informatics,Medical University Innsbruck, Innsbruck AT-6020, Austria; University Clinic of Internal Medicine III, Cardiology and Angiology, Medical University Innsbruck, Innsbruck AT-6020, Austria; University Clinic of Internal Medicine III, Cardiology and Angiology, Medical University Innsbruck, Innsbruck AT-6020, Austria; University Clinic of Internal Medicine III, Cardiology and Angiology, Medical University Innsbruck, Innsbruck AT-6020, Austria; University Clinic of Internal Medicine III, Cardiology and Angiology, Medical University Innsbruck, Innsbruck AT-6020, Austria; Department of Cardiology, HOCH Health Ostschweiz, Kantonsspital St. Gallen, St. Gallen CH-9007, Switzerland; Center for Health and Bioresources, AIT Austrian Institute of Technology, Graz AT-8020, Austria; Center for Health and Bioresources, AIT Austrian Institute of Technology, Graz AT-8020, Austria; Division of Cardiology, Department of Internal Medicine, Medical University Graz, Graz AT-8020, Austria; Division of Cardiology, Department of Internal Medicine, Medical University Graz, Graz AT-8020, Austria; Digital Cardiology Lab, University Clinic of Internal Medicine III, Medical University Innsbruck, Innsbruck AT-6020, Austria; University Clinic of Internal Medicine III, Cardiology and Angiology, Medical University Innsbruck, Innsbruck AT-6020, Austria; University Clinic of Internal Medicine III, Cardiology and Angiology, Medical University Innsbruck, Innsbruck AT-6020, Austria; University Clinic of Internal Medicine III, Cardiology and Angiology, Medical University Innsbruck, Innsbruck AT-6020, Austria

**Keywords:** Atrial fibrillation, Screening, Wearables, ECG, Digital health

Atrial fibrillation (AF) is common, frequently asymptomatic, and a major cause of stroke in older adults. Current guidelines recommend AF screening in individuals aged ≥65 years.^1,2^ As AF screening shifts towards scalable, remote, population-based strategies, self-applied ECG patch systems are increasingly incorporated into the diagnostic pathway.^3–7^ This reflects a shift in digital health, where parts of the diagnostic process are increasingly transferred from healthcare professionals to patients. Screening effectiveness therefore depends not only on the diagnostic performance of ECG technologies but also on whether older adults can successfully apply and activate these devices without supervision.

However, the older adults most likely to benefit from AF screening may face barriers to successful self-application because of age-related motor, visual, cognitive, and digital limitations.^8^ Despite widespread clinical use, most ECG patch systems have been evaluated under professional supervision or in selected populations, with little evidence on their independent, unsupervised use by older adults.

We conducted an investigator-initiated prospective randomized trial at the Medical University of Innsbruck, Austria (ClinicalTrials.gov, NCT07564349). Adults aged ≥65 years with an indication for ambulatory ECG monitoring were randomly assigned (1:1:1:1:1:1) to one of six commercially available CE-certified ECG patch systems. Allocation was concealed using sealed, identical envelopes prepared by study personnel not involved in device distribution. Participants received the assigned device with manufacturer-provided instructions only and applied and activated it at home without training or supervision. They were instructed to wear the device continuously for 7 days. The co-primary endpoints were the proportion of participants achieving ≥24 h of analyzable ECG and cumulative analyzable ECG-recording time. Secondary endpoints included need for assistance, patient-reported usability, and device-related adverse effects. Analyses followed the intention-to-treat principle. Between-group differences were assessed using Fisher's exact test for the binary endpoint and the Kruskal–Wallis test for cumulative ECG-recording time. The association between age and ≥24-h recording was explored using logistic regression adjusted for the assigned system. With 25 participants per group, the study had 80% power (two-sided α=0.025) to detect either a ≥ 24-hour analyzable ECG rate of ≤65% vs. 95% or a mean analyzable ECG recording time of <4.5 vs. 6 days. The study was approved by the Ethics Committee of the Medical University of Innsbruck, and all participants provided written informed consent.

Between September 2025 and May 2026, 321 individuals were screened, of whom 150 were randomized. Median age was 73.8 years (IQR 69.3–78.3), and 54 participants (36.0%) were women. Both co-primary endpoints differed significantly across systems (*P* < 0.001 for each). The proportion of participants achieving at least 24 h of analyzable ECG ranged from 24.0% to 76.0%, an absolute difference of 52 percentage points (95% CI 24.5–69.7) between the best- and worst-performing devices (*Figure 1A*). Median analyzable ECG-recording time ranged from 0 h in three of the six systems to 147 h in the highest-performing device (*Figure 1B*). Smartphone-dependent systems were among the lowest performers, required assistance more often, and showed lower recording success than standalone systems. Skin reactions and premature patch removal were uncommon across all groups. Older age was independently associated with a lower likelihood of achieving ≥24 h of analyzable ECG (odds ratio 0.92/year, 95% CI 0.86–0.98; *P* = 0.013). There was no association between educational status and outcomes (*P* = 1.00).

**Figure 1:**
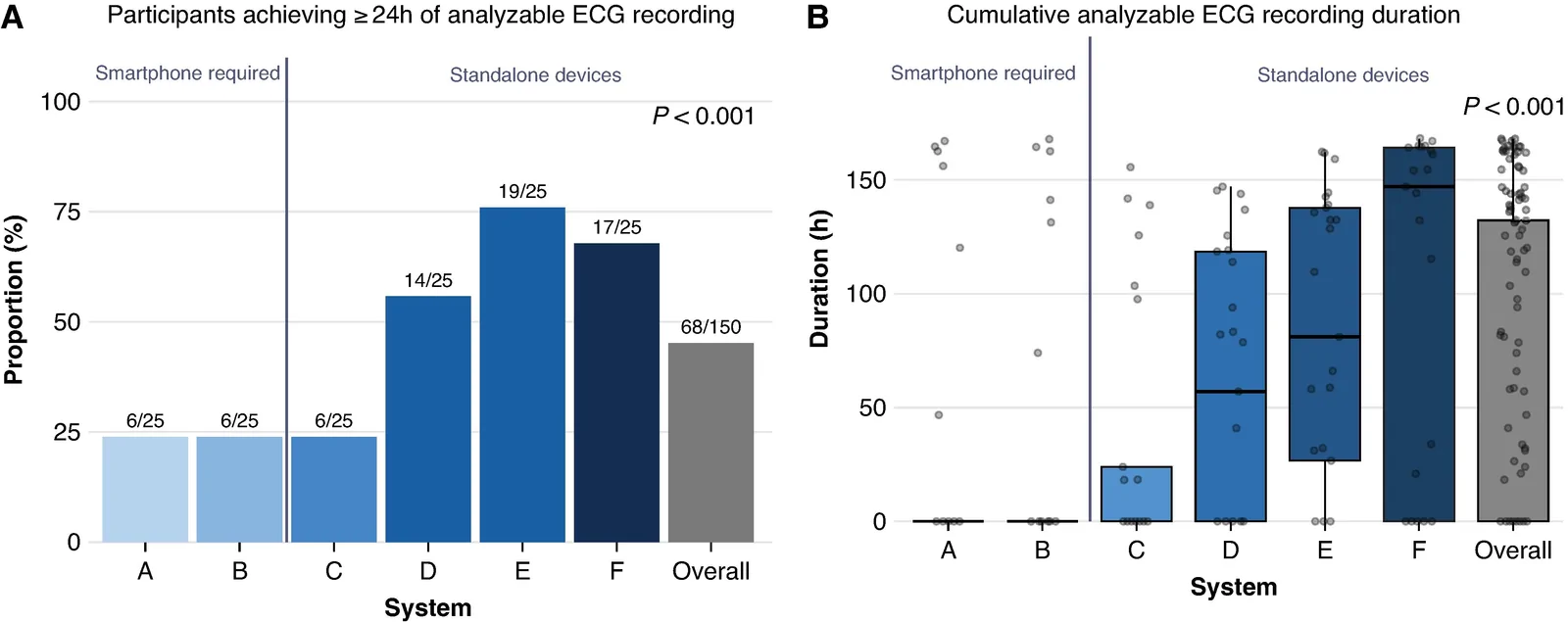
Co-primary endpoints by self-applied ECG patch system. **(***A***)** Proportion of participants achieving at least 24 h of analyzable ECG per system. **(***B***)** Cumulative analyzable ECG time per system, shown as the median with whiskers indicating the interquartile range. The vertical divider distinguishes systems requiring smartphone pairing (Smartcardia and ECG247) from standalone devices (Getemed, Kardiobeat, Biotronik, and ZIO). Systems: A—Smartcardia 7L (SmartCardia, Lausanne, Switzerland), B—ECG247 (Appsens, Lillesand, Norway), C—HeartX Recorder (Getemed, Teltow, Germany), D—Kardiobeat (Medicalgorithmics, Warsaw, Poland), E—net_ECG (Biotronik, Berlin, Germany), F—ZIO (iRhythm Technologies, San Francisco, California, USA). ECG = electrocardiography.

In this randomized comparison of six self-applied ECG patch systems, we observed marked differences in the amount of analyzable ECG obtained under unsupervised real-world conditions. However, the most striking finding was not the variation between systems but the low overall performance. Across all systems, only 68 of 150 participants (45.3%) achieved the predefined threshold of ≥24 h of analyzable ECG, while the median cumulative analyzable ECG recording time was only 0 h (IQR 0–132).

To our knowledge, this is the first randomized, head-to-head comparison of multiple commercially available, CE-certified ECG patch systems under unsupervised self-application by older adults. It is the first to show that the principal barrier to successful remote ECG monitoring lies before signal acquisition, at device application and activation, rather than in ECG signal quality or rhythm-detection accuracy. This identifies a previously underappreciated failure point in the diagnostic pathway of self-applied ECG monitoring.

These findings have direct implications for scalable remote AF screening strategies. As screening increasingly shifts from healthcare facilities to patients’ homes, successful device application can no longer be assumed but becomes a prerequisite for effective screening.^9^ Large-scale remote screening cannot rely on healthcare professionals to perform device application; instead, patients must be able to correctly attach, activate, and maintain the recording independently. Successful patient-performed ECG acquisition therefore becomes a prerequisite for any rhythm-detection algorithm, regardless of its diagnostic accuracy.

More broadly, our findings illustrate a fundamental challenge of digital health. As diagnostic processes are increasingly transferred from healthcare professionals to patients, usability becomes a determinant of diagnostic effectiveness rather than merely a convenience feature.^10^ Even highly accurate algorithms cannot compensate if the required diagnostic data are never successfully acquired.

The poor performance of smartphone-dependent systems, the inverse association between age and successful recording, and the absence of an association with educational status further support this interpretation. Performance under professional application or in digitally selected populations should therefore not be extrapolated to unsupervised self-use in older adults.

Several limitations should be acknowledged. The study was conducted at a single centre with a modest number of participants. The comparison between smartphone-dependent and standalone systems should be considered exploratory, as the systems differ in several design features beyond smartphone dependence. Furthermore, some evaluated systems were originally designed for application by healthcare professionals rather than for unsupervised self-use. Nevertheless, such systems are increasingly incorporated into remote diagnostic pathways in which independent application is often unavoidable, making evaluation under these conditions clinically relevant. Finally, this study assessed the feasibility of obtaining analyzable ECG recordings rather than AF detection or clinical outcomes.

In conclusion, commercially available self-applied ECG patch systems showed substantial differences in real-world performance among older adults, while overall performance was poor. These findings identify device application and activation as a critical determinant of the effectiveness of scalable remote AF screening strategies.

## Data Availability

The data underlying this article cannot be shared publicly due to the privacy of individuals who participated in the study. The data will be shared on reasonable request to the corresponding author, subject to applicable ethical and data protection requirements.
